# An Atypical Case of Moyamoya Disease With Recurrent Ischemic Events

**DOI:** 10.7759/cureus.81565

**Published:** 2025-04-01

**Authors:** Alan Rachid, Ameer A Khan, Thet Myat, Huma Khan, Mohammad Humeda

**Affiliations:** 1 General Internal Medicine, Tameside General Hospital, Ashton-Under-Lyne, GBR; 2 Cardiology, Tameside General Hospital, Ashton-Under-Lyne, GBR; 3 General Medicine, Leeds Teaching Hospital, Leeds, GBR; 4 Internal Medicine, Tameside General Hospital, Ashton-Under-Lyne, GBR; 5 General Medicine, Tameside General Hospital, Ashton-Under-Lyne, GBR

**Keywords:** cerebrovascular disorders, magnetic resonance imaging, moyamoya disease (mmd), multi-disciplinary team, neuro rehabilitation

## Abstract

Moyamoya disease (MMD), a progressive cerebrovascular disorder characterized by stenosis of intracranial arteries and the formation of compensatory collateral networks, presents complex management challenges. This case examines the clinical course of a Caucasian woman in her late 30s over four hospital admissions spanning less than a year, highlighting the interplay between medical, surgical, and rehabilitative interventions. It illuminates the multidisciplinary approach required to diagnose and manage MMD and its complications and the importance of investigating atypical presentations of the condition.

## Introduction

Moyamoya disease (MMD) is a rare, progressive cerebrovascular disorder characterized by stenosis or occlusion of the distal internal carotid arteries and their proximal branches, accompanied by the development of fragile collateral vessels, often described as a “puff of smoke” on angiography [1,2]. Diagnosis relies on magnetic resonance and computed tomography angiography of the cerebral vasculature capturing the steno-occlusive disease present [3]. This condition predominantly affects children and young adults, with a high prevalence in the East Asian population, leading to ischemic or hemorrhagic strokes, transient ischemic attacks (TIAs), and progressive neurological deficits [3]. MMD can be distinguished from Moyamoya syndrome, with the latter a consequence of causative diseases or associated conditions and MMD being idiopathic [3]. MMD in the Caucasian, western population is a rarity, with very few studies outside of Asia available to infer from [3].

While the pathophysiology remains incompletely understood, MMD is associated with both genetic and environmental factors, and its management often necessitates a multidisciplinary approach involving medical, surgical, and rehabilitative interventions [3].

This report presents the case of a 38-year-old female patient diagnosed with MMD following recurrent ischemic events, detailing her clinical course, including diagnostic findings, therapeutic strategies, surgical intervention, and outcomes. The case highlights the complexities of managing this rare condition, emphasizing the importance of individualized treatment plans and long-term surveillance.

## Case presentation

**Figure 1:**
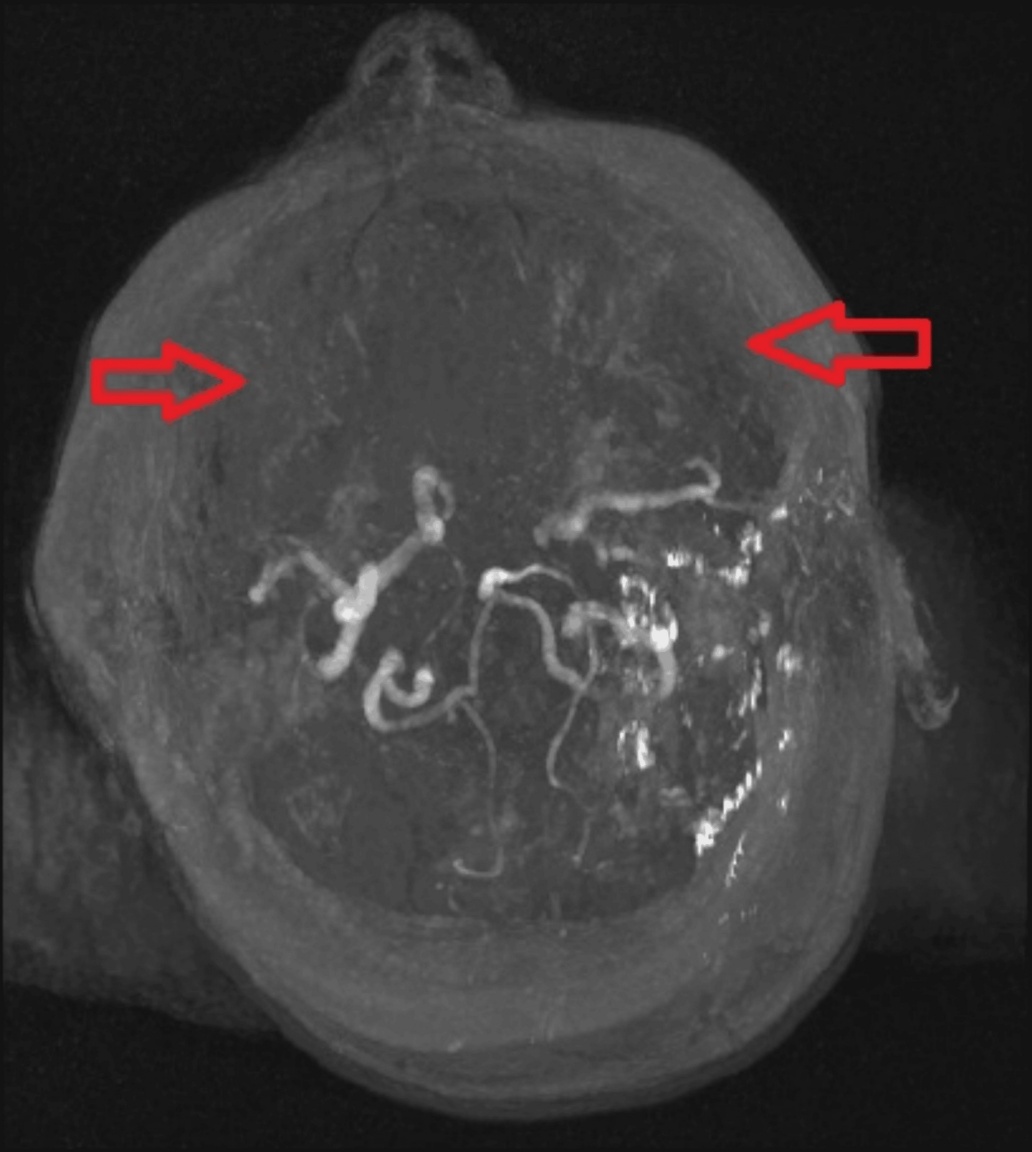
MRI imaging showing "puff of smoke" characteristic of Moyamoya disease

A 38-year-old Caucasian female patient presented in June 2023 with a one-week history of progressive expressive and receptive aphasia, culminating in right-sided numbness and an inability to speak. On admission, the NIHSS (National Institutes of Health Stroke Scale) was 1 with a Modified Rankin Score (mRS) of 1. Neuroimaging identified a small acute infarct in the left frontal cortical region within the middle cerebral artery (MCA) territory. Subsequent MRI and MRA confirmed multifocal infarcts with vascular changes characteristic of MMD (Figure 1). Initial management included the initiation of antiplatelet therapy (aspirin transitioned to clopidogrel for secondary prevention), atorvastatin for dyslipidemia, and speech and occupational therapies. The patient demonstrated moderate recovery and was discharged with plans for follow-up in a young stroke clinic and further evaluation to elucidate the underlying etiology. An echocardiogram was subsequently performed and was normal. Autoimmune, thrombophilia, and antiphospholipid screening were negative. 

**Figure 2:**
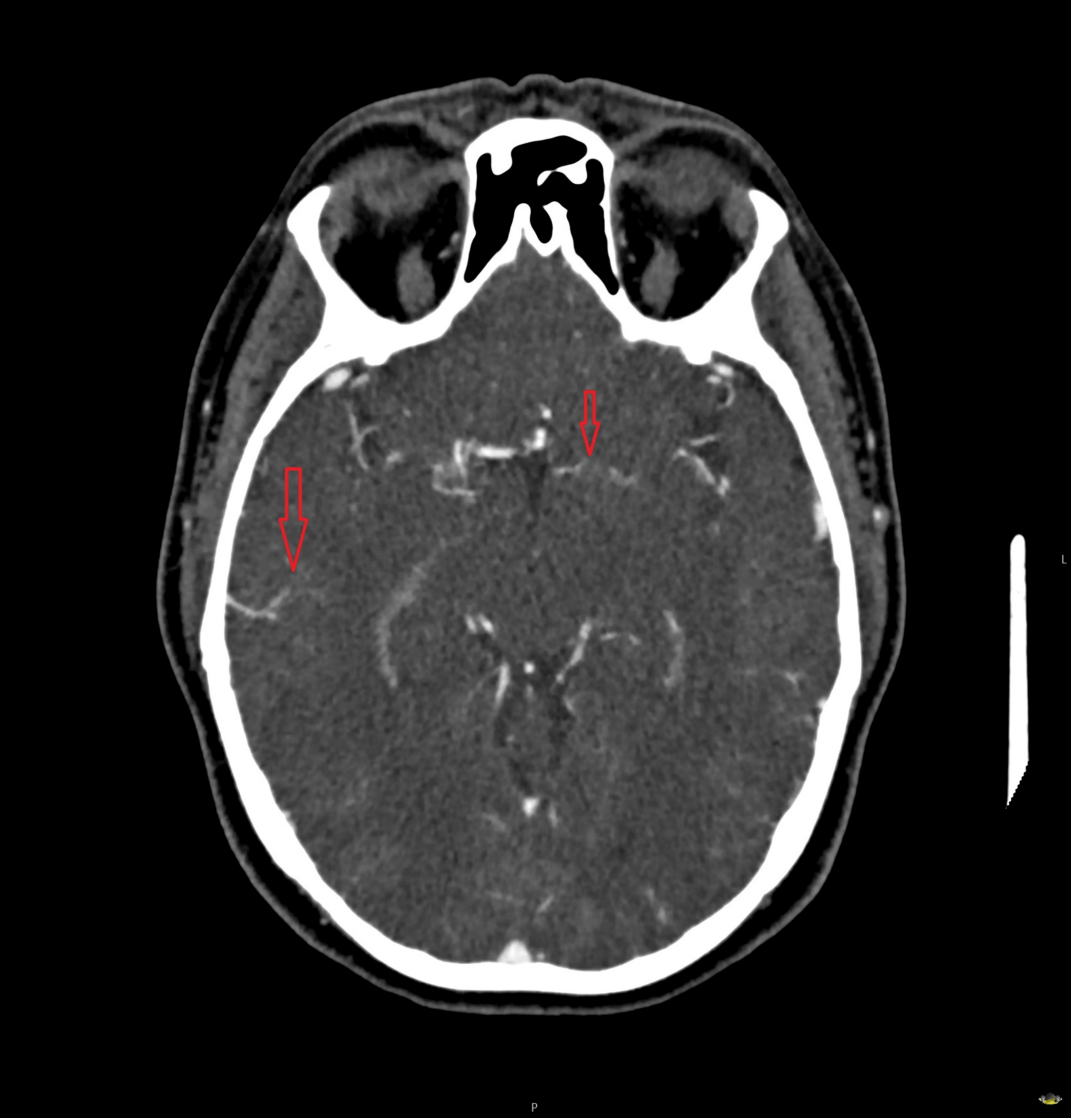
CT imaging showing early signs of Moyamoya disease with collateral vessel formations

**Figure 3:**
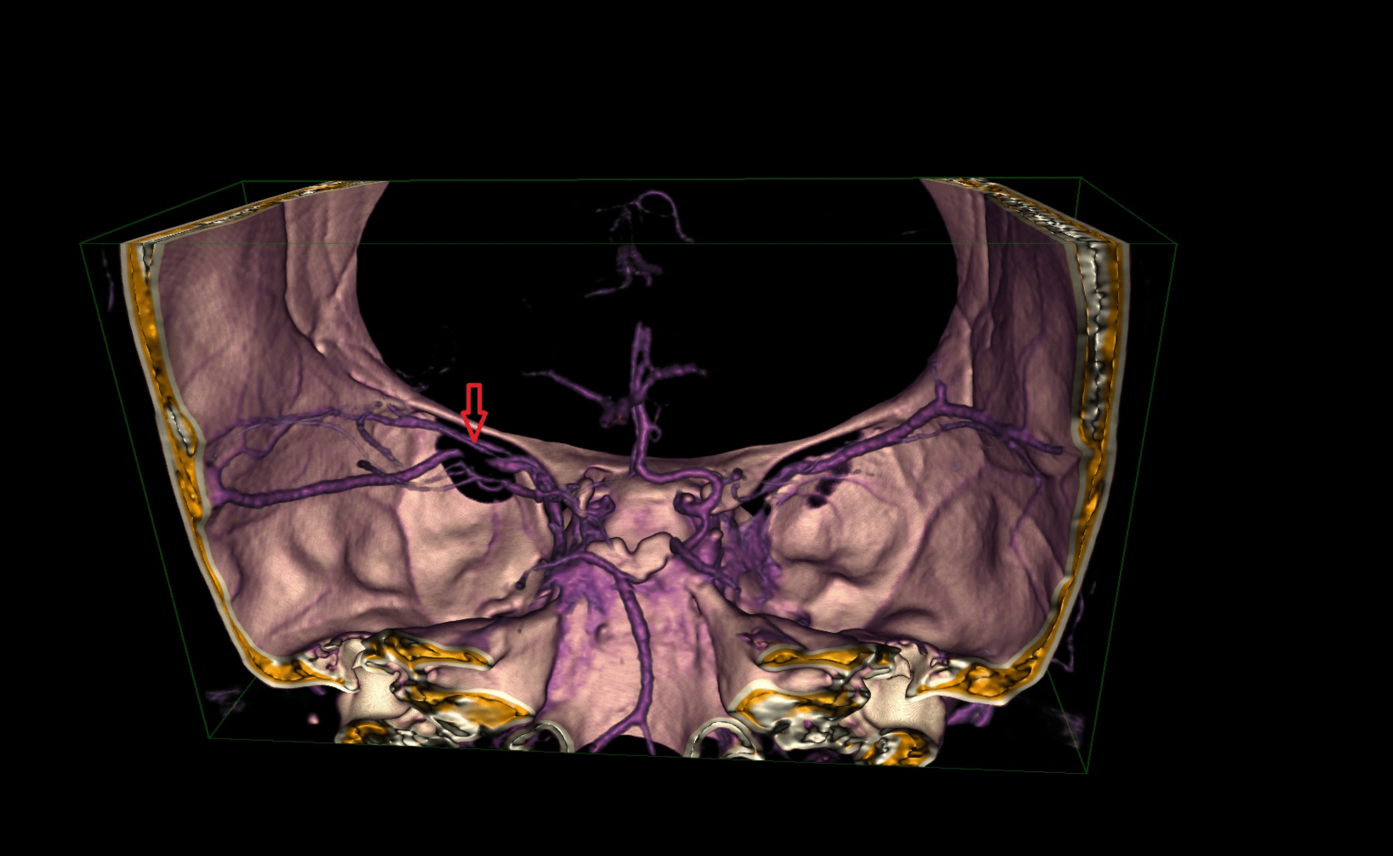
CT imaging in 3D demonstrating stenosis

Shortly after discharge, in July 2023, the patient was readmitted with auditory disturbances and transient speech issues. Later that month, she experienced a TIA with transient right-sided weakness and speech disturbances. Imaging revealed progressive stenosis and collateral vessel formation typical of MMD (Figure 2 and Figure 3). Dual antiplatelet therapy (aspirin and clopidogrel) was maintained, alongside strategies to optimize cerebral perfusion, including hydration and blood pressure management. A multidisciplinary neurovascular team discussed revascularization options, such as direct bypass surgery and encephaloduroarteriosynangiosis (EDAS). Following stabilization, she was discharged with plans for continued neurovascular follow-up.

In November 2023, the patient underwent extracranial-to-intracranial (EC-IC) bypass surgery to improve cerebral perfusion. Postoperatively, she developed complications, including vasospasm in the left MCA, resulting in transient unresponsiveness, right-sided weakness, and dysphasia, necessitating admission to critical care. Management included the MEND protocol, with nimodipine and metaraminol infusions to augment blood pressure (target MAP >90 mmHg) and aspirin for anticoagulation. Neurological monitoring detected new ischemic strokes, though gradual improvement in motor strength and speech clarity was noted. She was discharged in early December 2023 with plans for outpatient rehabilitation and continued monitoring.

Four months post-surgery, in March 2024, the patient presented with an ischemic stroke attributed to missed doses of clopidogrel. At this point, an NIHSS of 6 and an mRS of 1 were recorded. Clinical features included mild dysarthria, right-sided weakness, and sensory deficits. Cilostazol was initiated as an alternative antiplatelet agent due to its efficacy in MMD, and neurosurgical evaluation ruled out the need for immediate intervention. The patient recovered significantly and was discharged with reinforced counseling on medication adherence.

Later in March 2024, the patient experienced acute neurological deterioration characterized by right-sided weakness, dysarthria, and altered consciousness. On this admission, the NIHSS was recorded as 20 and the mRS as 2. Myoclonic seizure activity was observed at the time, though upon review by the neurology team this was deemed secondary to cerebral irritation caused by an extensive ischemic event. Imaging demonstrated ischemic changes in the right frontal and parietal regions. Management included the initiation of levetiracetam to control focal seizures, blood pressure stabilization with amlodipine and doxazosin, and pain management with morphine. Family discussions emphasized the progressive nature of MMD and the limited prospects for further surgical intervention. Supportive care focused on quality of life, and the patient stabilized sufficiently for transfer to a specialized rehabilitation facility. She remained with persistent neurological deficits but exhibited improved comfort and seizure control. A summarized timeline of events is shown in Table 1.

**Table 1:** Timeline of events that occurred during multiple admissions NIHSS:  National Institutes of Health Stroke Scale; mRS: Modified Rankin Score; MEND: Metabolic Enhancement for Neurodegeneration

Date	Event	Intervention/Management	Patient Deterioration
June 2023	Initial presentation with aphasia, right-sided numbness, and inability to speak. NIHSS = 1, mRS = 1	Initiated antiplatelet therapy (aspirin, later clopidogrel), atorvastatin, speech/occupational therapies.	Mild symptoms; NIHSS and mRS scores indicate minimal functional impairment.
July 2023	Readmission with auditory disturbances, transient speech issues, and transient ischemic attack (TIA).	Dual antiplatelet therapy (aspirin + clopidogrel), optimization of cerebral perfusion (hydration, blood pressure management).	Progression of symptoms: transient right-sided weakness, speech disturbances. Imaging reveals stenosis.
July 2023	Multidisciplinary team discusses revascularization options (EC-IC bypass, EDAS).	Continued neurovascular follow-up.	Progressive stenosis and collateral vessels. No immediate intervention but ongoing monitoring.
November 2023	EC-IC bypass surgery for cerebral perfusion improvement. Postoperative complications.	Post-op care with MEND protocol, nimodipine and metaraminol for vasospasm, aspirin for anticoagulation.	Transient unresponsiveness, right-sided weakness, dysphasia due to vasospasm. New ischemic strokes detected.
December 2023	Discharged after surgery with plans for rehab and monitoring.	Outpatient rehab and continued neurovascular follow-up.	Gradual neurological improvement post-surgery.
March 2024	Ischemic stroke due to missed clopidogrel doses. NIHSS = 6, mRS = 1.	Initiated cilostazol as an alternative antiplatelet agent. Neurosurgical evaluation to rule out immediate intervention.	Mild dysarthria, right-sided weakness, and sensory deficits. Significant improvement noted after treatment initiation.
March 2024	Acute neurological deterioration: right-sided weakness, dysarthria, altered consciousness. NIHSS = 20, mRS = 2	Started levetiracetam for seizures, blood pressure management (amlodipine, doxazosin), pain management (morphine).	Severe deterioration: myoclonic seizures, significant ischemic changes, and impaired consciousness.
March 2024 (Later)	Transfer to rehabilitation facility after stabilization.	Supportive care focusing on quality of life, seizure control, and comfort.	Persistent neurological deficits but improved comfort and seizure control post-intervention.

## Discussion

The clinical manifestations of MMD are broad and complex, which further highlights the rarity of the condition. There have been numerous revisions of the guidelines and varying criteria needed to help establish an MMD diagnosis [3,4]. Previous criteria focused on bilateral occlusive changes in the internal carotid arteries; however, there is now scope for patients with unilateral ICA occlusive disease to be considered for MMD [3].

As demonstrated in this case, MMD has a profound impact on a patient’s quality of life and a multidisciplinary approach is vital to yield the best outcome. The psychosocial burden of MMD is substantial, affecting both patients and their families, yet it is often underestimated. Given the unpredictable nature of the disease and its potential to cause recurrent ischemic events and associated disabilities, patients with MMD may experience significant psychological distress, including anxiety and depression [5]. The resultant loss of independence and significant changes in their social roles can further contribute to feelings of isolation and helplessness. Additionally, family members often experience emotional strain and exhaustion, which highlights the importance of psychological support and counselling in order to address the psychosocial needs of both patients and families.

The paucity of data in and around MMD, particularly in populations outside of Eastern Asia, further complicates the ability for clinicians to make accurate diagnoses [3]. However, advancements in neuroimaging have enabled earlier and more accurate detection of the disease, which is crucial for timely intervention. Despite these advancements, the progressive nature of MMD still often results in a recurrent ischemic phenomenon, which underscores the crucial need for preventive measures to reduce such occurrences.

As is the nature of MMD, the recurrent ischemic phenomenon continues to occur in these patients and preventive strategies are vital to help this at-risk population. Medical management primarily focuses on symptom control and prevention of complications. Antihypertensive medications, including calcium channel blockers and antiplatelet therapy such as aspirin, are commonly used to maintain cerebral perfusion and reduce the risk of thromboembolic events but are not always sufficient, particularly in cases with significant ischemic symptoms or recurrent strokes [6].

Surgical options, as considered in this case, theoretically help to improve cerebral perfusion and revascularization [4]. High-resolution MRI has been investigated as a tool to capture early angiographic findings of MMD [3]. Revascularization procedures, such as superficial temporal artery to middle cerebral artery bypass, remain fundamental in improving cerebral perfusion of the brain [7,8]. The decision to proceed with surgical intervention depends on multiple factors including age, clinical presentation, and overall health status [6,7]. Although surgical treatment options are associated with favorable outcomes, risks such as infection and anesthesia-related complications must be carefully considered. In some cases, a combination of medical and surgical therapy is needed to achieve optimal outcome [6].

A multidisciplinary team, involving neurologists, neurosurgeons, rehabilitation specialists, and mental health professionals, is essential in comprehensive management of MMD ranging from acute medical management to long-term rehabilitation and psychosocial support [6,8]. Regular follow-ups and monitoring are crucial to assess disease progression and efficacy of treatment. In addition, providing education and support to families empowers them to actively participate in patient care, which in turn improves the overall management and long-term outcomes [6].

## Conclusions

This case illustrates the complexities inherent in managing MMD, particularly in cases with recurrent ischemic events and complications from surgical interventions. Through the use of a multidisciplinary approach, including revascularization surgery, antiplatelet therapy, and comprehensive rehabilitation, care was tailored to optimize functional recovery and prevent further vascular events. This case underscores the importance of personalized treatment strategies, vigilant monitoring, and patient education to improve long-term outcomes in MMD patients. When an MMD patient continues to have ischemic events, consideration should be given to early surgical input. This also highlights the need to ensure physicians maintain an awareness of atypical presentations to help them yield early diagnoses of this subset of patients. There was an absence of pediatric onset in this case as well as it being seen in the Caucasian population, both atypical of MMD. 
